# Mechanical characterization and constitutive law of porcine urethral tissues: a hyperelastic fiber model based on a physical approach

**DOI:** 10.1007/s10237-025-01951-w

**Published:** 2025-04-10

**Authors:** Quentin De Menech, Andres Osorio Salazar, Quentin Bourgogne, Yoan Civet, Adrien Baldit, Yves Perriard

**Affiliations:** 1 Integrated Actuators Laboratory (LAI), Ecole polytechnique fédérale de Lausanne (EPFL), Neuchâtel, 2002 Switzerland; 2 ENIM, Université de Lorraine, Metz, 57000 France; 3 Université de Lorraine, CNRS, LEM3, Metz, 57000 France

**Keywords:** Anisotropy, Biomechanics, Ex vivo testing, Hyperelastic models, Urinary incontinence

## Abstract

Lower urinary tract symptoms (LUTS), particularly urinary incontinence (UI), represent a significant global health challenge, affecting millions of patients worldwide. The artificial urinary sphincter (AUS) remains one of the most effective intervention for severe UI, with its design relying on a detailed understanding of the urethral biomechanics. Given the ethical and logistical constraints of using human tissue, porcine urethras, which share anatomical and mechanical similarities with human urethras, are widely employed in preclinical studies. This study investigates the uniaxial mechanical characterization of porcine urethral tissue under controlled conditions. Fresh porcine urethral samples were subjected to uniaxial tensile testing along both the longitudinal and circumferential directions to characterize their anisotropic mechanical properties. Experimental results were compared with existing datasets to validate findings. Additionally, conventional hyperelastic models were assessed to fit experimental results, and a novel anisotropic constitutive model with physical parameters was developed. This fiber model, which incorporates fiber modulus, volume, and orientation, uses a single set of parameters to predict behavior in both directions. It demonstrated improved accuracy, reaching the performance of the Gasser-Ogden-Holzapfel (GOH) model, with root mean square errors (RMSEs) of 9.24% and 12.98% in the circumferential and longitudinal directions, respectively. In contrast, the Yeoh and Ogden models were unable to fit both directions using a single set of parameters, yielding RMSEs values exceeding 30%. With its enhanced physical relevance, the fiber model having a more physical meaning holds promise for applications in the biomechanical analysis of fiber-composed soft tissues.

## Introduction

The urethra plays a critical function in the urinary system, with its mechanical characteristics directly impacting physiological processes during micturition O’Meara et al. (2024); Natali et al. (2016); Schäfer (1985). Consequently, understanding the mechanical behavior of urethral tissues is essential for addressing a range of urological conditions Norton and Brubaker (2006); Yu et al. (2013); Jankowski et al. (2006). Accurately characterizing the mechanical properties of these tissues is critical for the development of effective treatments and surgical interventions. Advancements in biomedical engineering have underscored the importance of detailed mechanical analyses to enhance our knowledge of soft tissue mechanics Cowin and Doty (2007); Guimarães et al. (2020); Yc (2013).Despite these advancements, significant knowledge gaps remain in the understanding of the urinary tract, particularly the urethra Pipitone et al. (2021). A thorough comprehension of urethral mechanics is essential for improving the management of lower urinary tract dysfunctions and symptoms (LUTS), such as benign prostatic hyperplasia Chughtai et al. (2016) and urinary incontinence Nambiar et al. (2018); Aoki et al. (2017). Moreover, this understanding is critical for optimizing the design of medical devices that interact with the urethra, including stents Sali and Joshi (2020); Ramachandra et al. (2020); Haleblian et al. (2008), catheters Newman et al. (2017); Feneley et al. (2015), and artificial urinary sphincters (AUS) Peyronnet et al. (2020); Van der Aa et al. (2013); De Menech et al. (2024b). Our research is motivated by the need to improve treatments for patients suffering from urinary incontinence. We are developing an Artificial Urinary Sphincter (AUS) to help restore normal bladder control in affected individuals. To develop an effective AUS, it is crucial to gain a comprehensive understanding of urethral mechanical behavior. Despite its clinical importance, studies on the mechanical behavior of human urethral tissue remain limited, primarily due to ethical and logistical challenges Versteegden et al. (2017); O’Meara et al. (2024). Consequently, much of the research in this area has been conducted on animal models Feng et al. (2010); Shen et al. (2021). The most comprehensive mechanical data on urethral tissue, particularly from large animal models, have been provided by Natali et al. (2016); Natali et al. (2017a, 2017b), who investigated the mechanical properties of horse urethra. More recently André et al. (2022) demonstrated that while the porcine urethra shares certain mechanical characteristics with the human urethra, notable differences still exist. Similarly, Muller et al. (2008); De Menech et al. (2023) characterized the mechanical properties of the sow urethra, presenting differing results. Among animals, the porcine urethra remains the most suitable model due to its mechanical behavior and geometry, which resemble those of the human urethra. Masri et al. (2018); Cunnane et al. (2021b); Ragionieri et al. (2016); Cartes et al. (2025); Berardo et al. (2024). However, disparities exists in literature between experimental results, additional mechanical testing on porcine urethra is necessary to enhance confidence in the data and to further solidify its relevance as a replacement to human urethra during preclinical studies. Furthermore, an in-depth understanding of urethral tissue at a microscopic level, particularly its spatial organiization, fiber geometry and histological composition of its layers is essential for accurately modeling its mechanical properties. Histological analysis provides critical insights into the orientation and distribution of fibers and the structural arrangement of each layer, enabling us to make informed assumptions about material symmetry. The work of Ragionieri et al. (2016); De Menech et al. (2024a) as well as Hinata et al. (2013) has been referenced to define the microstructure of the urethra in the section 2.3. Furthermore, several studies have employed hyperelastic models to characterise the mechanical behavior of soft biological tissues Chagnon et al. (2015); Martins et al. (2006), including the urethra Masri et al. (2018). These models capture the nonlinear stress–strain response by defining a strain energy density function. They applied a variety of hyperelastic models, including Ogden model Ogden (1997); Ogden et al. (2004) and Yeoh model Yeoh (1993); Rivlin (1997) to fit experimental data for porcine urethra. Similarly, Masri et al. (2018) applied the GOH model Gasser et al. (2006); Holzapfel et al. (2000) to capture the anisotropic behavior of human urethral tissue. Existing hyperelastic models have significantly advanced the predictive capabilities of soft tissue mechanics, providing valuable insights into tissue behavior under loading conditions. However, most existing models remain predominantly empirical (Neo-Hookean, Ogden, Yeoh), relying on curve-fitting techniques rather than explicitly incorporating microstructural parameters Yeoh (1997); Wex et al. (2015); Przybylo and Arruda (1998). Despite progress in material science, fully characterizing hyperelastic materials remains challenging due to the inherent complexity of their microstructural behavior. Many conventional models fail to account for key fiber-related parameters, such as fiber volume fraction, orientation distribution, and the mechanical contrast between fibers and the surrounding matrix Jor et al. (2013); Koponen (2007). Since fibers play a crucial role in the anisotropic response of biological tissues, this omission limits the models’ ability to accurately describe urethral tissue mechanics and reduces their generalizability across different tissue types and experimental conditions Xiang et al. (2019). To address these limitations, theoretical models would be the most appropriate. However, their applicability is often constrained by incomplete knowledge of the microstructural organization and mechanical interactions within soft tissues. As a result, a semi-empirical approach remains necessary to integrate both theoretical insights and experimentally derived parameters. In this study, we propose a semi-empirical hyperelastic model that explicitly incorporates fiber contributions in soft tissues. By integrating parameters related to fiber contribution in the mechanical behavior, our model provides a more physically informed representation of urethral tissue mechanics. This approach enables a more accurate description of the anisotropic behavior of urethral tissue in both the circumferential and longitudinal directions. Building on the need for more experimental data on porcine urethra and the development of a physically informed hyperelastic model, this paper is structured as follows: First, the anatomical characteristics of urethral tissue are described, along with the urethral sample extraction procedure, histological analysis, and symmetry assumptions. This is followed by a detailed explanation of the uniaxial tensile testing protocol used to obtain stress–strain relationships in both circumferential and longitudinal orientations. Next, existing hyperelastic models are introduced, followed by our proposed fiber-based model, which explicitly incorporates physically parameters associated with fiber contribution. Experimental results are then compared with previously reported data on porcine urethra from André et al. (2022) and human urethra from Masri et al. (2018). A comparative analysis of hyperelastic model fitting is conducted, including our fiber-based model, with root mean square error (RMSE) values reported to evaluate predictive performance. The objectives of this study are twofold: (1) to compare the mechanical behavior of porcine urethral tissue in the circumferential and longitudinal directions with existing literature data, including human urethral tissue, and (2) to develop and assess the predictive performance of a novel hyperelastic model incorporating physical fiber-based parameters to better capture the anisotropic mechanical behavior of urethral tissue.

**Fig. 1:**
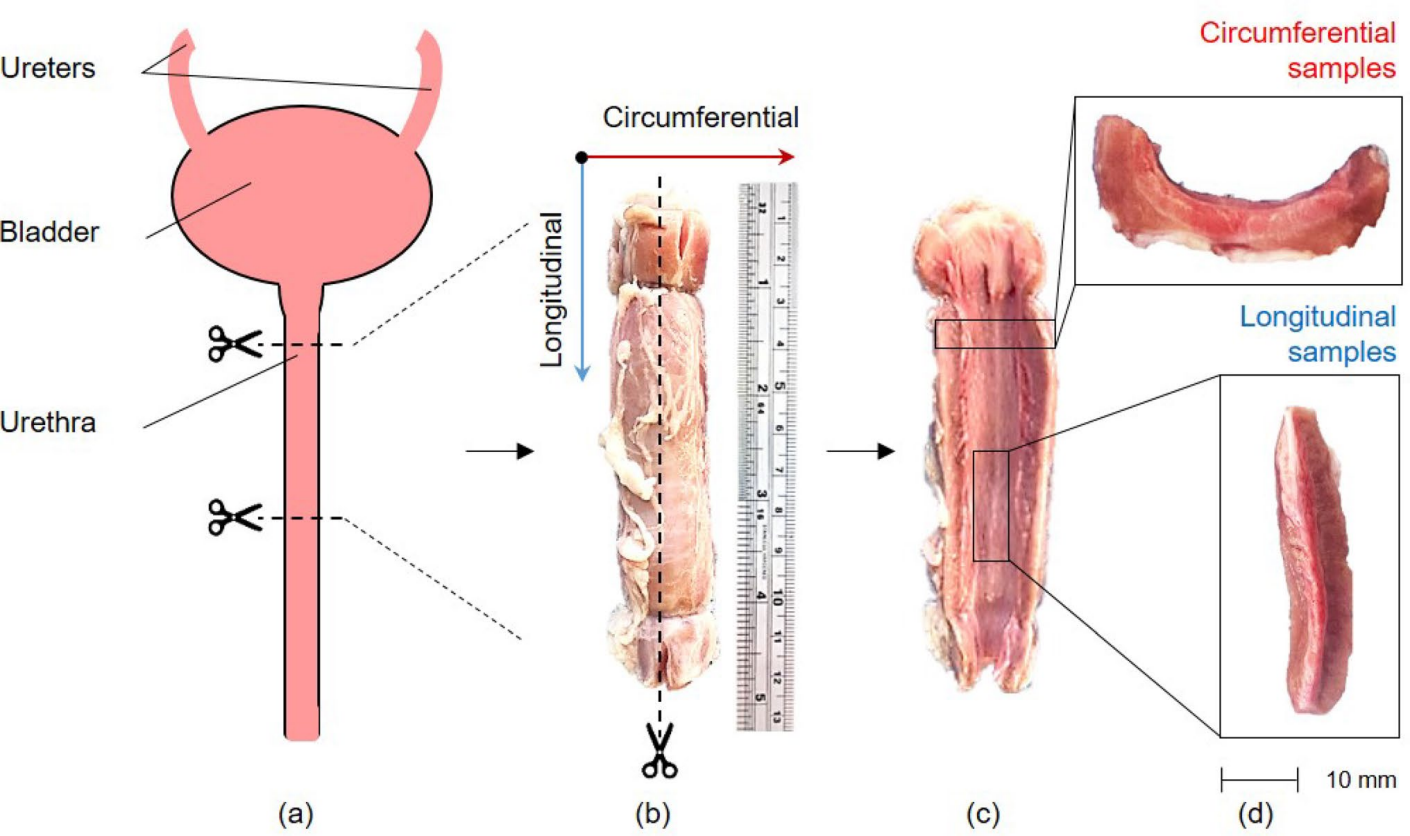
(a) Porcine urinary tract, (b) Urethra extracted from urinary tract, (c) Urethra after longitudinal incision, (d) Circumferential and longitudinal samples extracted

## Material and methodology

In this section, we describe the anatomical features of the urethra as well as explaining the sample extraction. Then, we propose a histological analysis from literature to understand the microstructure of the human urethra. This analysis enables us to make assumptions about material symmetry, which is critical for defining our hyperelastic model based on fiber orientation and geometry. Finally, we detail the mechanical testing procedure used to define the mechanical properties of our urethral samples.

### Anatomical description of porcine urethral tissue

The porcine urinary tract, as illustrated in Fig. 1 (a) extends from the internal urinary bladder orifice to the external opening. The urethra is divided into distinct regions based on functional and anatomical characteristics. The proximal section, located near the bladder, transitions from the bladder neck through the urethral sphincter, which acts as the primary control point for urine flow. This segment, analogous to the pre-prostatic urethra in humans, measures approximately 1 cm. Next, a middle portion of about 3-4 cm long, through which the urethra passes without direct contact with any glandular structures, differing from the human prostatic urethra. Following this, the membranous region, representing the least distensible part of the urethra, is identified. Finally, the distal urethra is distinguished by longitudinally aligned fiber tissue and a slight curvature with a ventral orientation relative to the animal’s anatomy. In this study, tissue samples were extracted from the distal region, as this area is analogous to the section studied by Masri et al. (2018). and corresponds to the optimal location for implanting an Artificial Urinary Sphincter Elliott and Barrett (1998); Brant and Martins (2017).

### Sample extraction

Three porcine female specimens were obtained from a certified slaughterhouse in Neuchâtel, Switzerland, in accordance with ethical and regulatory guidelines for the handling of animal tissues. Immediately post-slaughter, the specimens were placed in 0.9% saline solution preheated to a temperature of $36.5 \pm 1^{\circ }$C and stored in insulated containers to preserve tissue integrity Cunnane et al. (2021b); Johannessen et al. (2004). The animals, aged 6-8 months and with a body mass of approximately 90-110 kg, were selected to ensure consistency in tissue properties. For dissection, the entire urinary tract was provided for each animal. Three urethral samples were obtained from each specimen, each urethra have been cut at a distance of $20 \pm 5$ mm from the bladder neck and cut to a length of $120 \pm 10$ mm. The dissection process ensured clean cuts and intact tissue for mechanical testing. As shown in Fig. 1(b) and Fig. 1(c), the samples were first divided along the longitudinal axis. Smaller segments were then extracted in both longitudinal and circumferential directions (Fig. 1(d)). A total of six samples were prepared per urethra: three longitudinal and three circumferential, yielding a total of 18 samples. Immediately following excision, the samples were submerged in the saline solution to maintain hydration and prevent desiccation prior to testing.

### Histological analysis & symmetry assumption

**Fig. 2:**
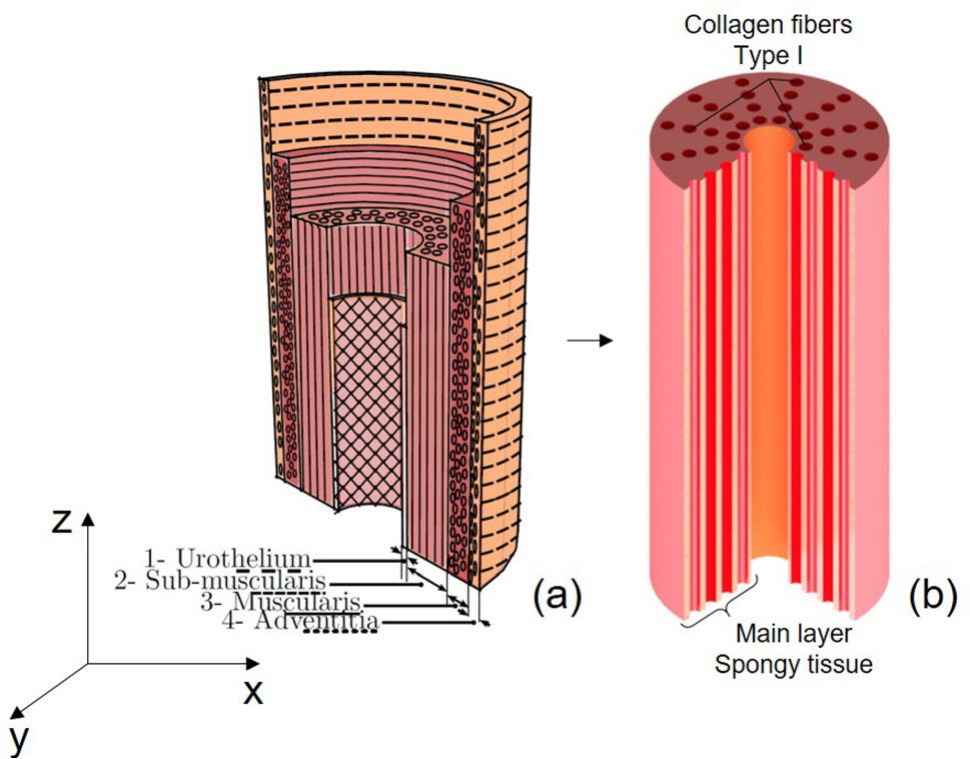
(a) 3D representation of a human urethra figure adapted from Masri et al. (2018) (b) Simplified representation of a human urethra considering one layer of submuscularis with collagen fibers

In order to define a hyperelastic model based on fiber orientation and geometry, it is necessary to detail with accuracy the microstructure of the urethra and therefore perform a histological analysis. We used the work previously done by Masri et al. (2018). to bypass the need for physical histological cuts while still capturing the critical microstructural details required for our analysis and hyperelastic model. The male human urethra, as shown in Fig. 2, is composed of four distinct layers. The innermost layer, known as the urothelium, consists of connective tissue containing capillaries and measures approximately 50 $\mu$m in thickness. The next layer, referred to as the submuscularis, is characterized by spongy tissue with vascular spaces and longitudinal smooth muscle fibers. These muscle fibers are organized into bundles, interspersed with numerous collagen and elastic fibers arranged in a circular pattern, forming the thickest layer of the urethra at approximately 100 $\mu$m. The external muscularis constitutes the third layer, measuring about 80 $\mu$m and composed of thin, discontinuous muscle cells arranged circularly. This layer is accompanied by a rich extracellular matrix containing circular collagen fibers. Finally, surrounding these structures is the adventitia, composed of loose connective tissue containing vascular and nervous elements. Immunohistochemical analysis reveals that the overall tissue is abundant in type I collagen fibers, which contribute to its structural integrity. The work from Dutov et al. (2016) reported a Young’s modulus for type I collagen in the range of 100 MPa to 360 MPa, with a mean value of $E_f$ = 230 MPa representing the stiffness of the fibers. The fiber volume fraction $\phi$ which is the volume fraction of fibers in the tissue compared to the matrix, derived from several studies in literature Ragionieri et al. (2016); Hinata et al. (2013); Žiaran et al. (2017) and is set at $\phi$ = 20%. Those values will be used to develop our fiber model later on in section 3.2. Considering the previous work by Masri et al. (2018), we assumed that, as shown in Fig. 2(b), the urethra consists of spongy tissue associated with longitudinal smooth muscle fibers composed of type I collagen. Based on this assumption, the urethra exhibits transversely isotropic behavior due to the alignment of collagen fibers along its longitudinal axis Ding et al. (2006). This assumption of transverse isotropy is sufficient for our case and simplifies the constitutive modeling described later in Sect. 2.5.

**Fig. 3:**
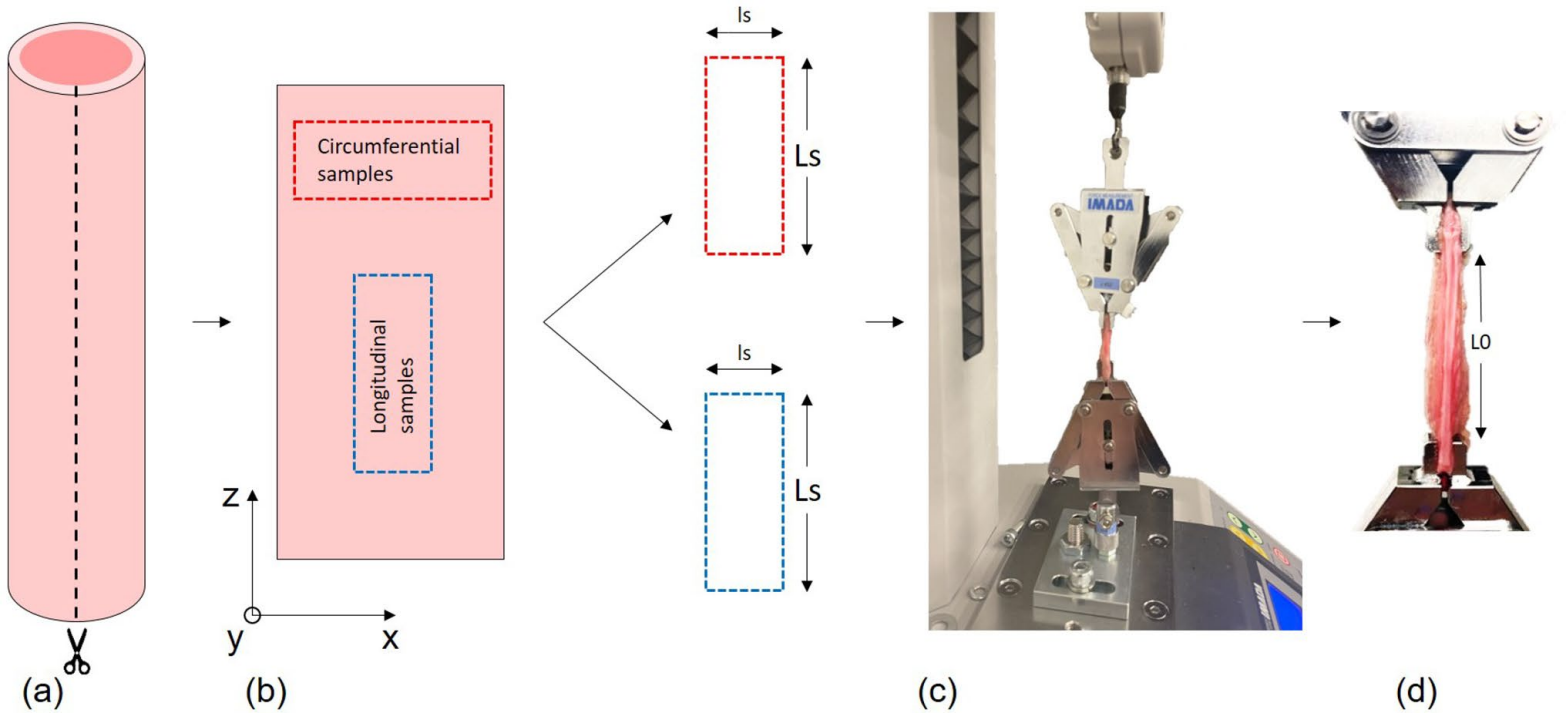
Tensile test experimental methodology. (a) Urethra cut along its longitudinal direction. (b) Planar specimens (circumferential and longitudinal) extracted from the unfolded urethra. (c) Specimen clamped at both extremity at an initial length $L_0$

### Mechanical tests

Mechanical characterization of the urethral tissue was conducted using a uniaxial tensile testing machine to assess its response under applied loads. To ensure results that closely replicate in vivo conditions, all samples were tested within two hours postmortem Van Ee et al. (2000). Samples were tested randomly to prevent selection bias and ensure against accidental bias Suresh (2011). During testing, urethral samples were maintained in saline solution at a temperature of $36.5 \pm 1^{\circ }$C and removed only for testing, with each test lasting a maximum of 2 min to minimize tissue dehydration. Samples were first cut longitudinally, as shown in Fig. 3(a), after which both circumferential and longitudinal samples were extracted, as depicted in Fig. 3(b). All samples had similar dimensions of $Ls$ = $40 \, \text {mm} \pm 0.5 \, \text {mm}$ and $ls$ = $8 \, \text {mm} \pm 0.5 \, \text {mm}$. Both circumferential and longitudinal samples were tested to investigate the anisotropic mechanical behavior of the urethral tissue.

Tensile testing was performed using an Imada^TM^ EMX tensile testing machine (Fig. 3(c)), equipped with a ± 20 N load cell. Load and displacement data were recorded throughout the tests. To ensure consistent testing conditions, each sample was mounted in a rigid frame using clamps specifically designed to prevent slippage and preserve tissue shape during mechanical loading. The clamps secured the samples at a distance of 5 mm from each extremity, resulting in an initial length $L_0$ = $30 \, \text{ mm } \pm 0.5 \, \text {mm}$ used to calculate the nominal strain. The potential influence of end effects due to the grip-to-grip length measurement approach must be acknowledged. We considered the crude estimation method proposed by Anssari-Benam et al. (2012). While an exact quantification was not performed due to the lack of data on the urethral shear modulus, a preliminary analysis suggests that end effects are likely to be minimal, given the aspect ratio of 3.75.

For each urethra, three samples were tested in each orientation (circumferential and longitudinal). Each sample was loaded to its fracture point. To ensure the accuracy of the data and prevent the inclusion of undesirable behavior, tests were excluded if failure or slippage was observed near the clamping region. Furthermore, due to the reduction in the urethra’s cross-sectional area beyond 0.6 strain, which could not be measured accurately, we adopted a conservative approach by focusing on the data within the 0.5 strain range. Consequently, two circumferential and two longitudinal samples per urethra were retained for analysis, yielding a total of 12 valid measurements out of the 18 tested samples.

Due to challenges in accurately measuring the cross-sectional area change over time during testing, we defined the strain and stress using nominal values. The nominal strain, $\varepsilon _n$ (dimensionless), is calculated as:

1 $$\begin{aligned} \varepsilon _n = \frac{L - L_0}{L_0} = \frac{\Delta L}{L_0} \end{aligned}$$

Here, $L_0$ is the original length, and *L* is the current length of the sample that increases over time during testing. The nominal stress, $\sigma _n$ (kPa), for a given applied force *F* and initial cross-sectional area $A_0$, is expressed as:

2 $$\begin{aligned} \sigma _n = \frac{F}{A_0} \end{aligned}$$

Knowing the speed at which tensile tests have been performed, one can define the strain rate as:

3 $$\begin{aligned} \dot{\varepsilon }_n = \frac{d}{dt} \left( \frac{L - L_0}{L_0} \right) = \frac{\dot{L}}{L_0} \end{aligned}$$

where $\dot{\varepsilon }_n$ is the strain rate, $\dot{L}$ is the rate of change of the length, and $L_0$ is the original length of the sample.

The tests were conducted at a speed of 0.83 mm/s. Given the initial sample length $L_0$, this corresponds to a strain rate $\dot{\varepsilon }_n = 2.8 \times 10^{-2} \, \text {s}^{-1}$. This strain rate was set to remain within the quasi-static domain Comley and Fleck (2012); Natali et al. (2016), minimize the influence of viscous phenomena Bagi et al. (2002); Thind (1995), and prevent dynamic effects that may arise at higher strain rates Yc (2013); Weiss and Gardiner (2001). Additionally, it ensured a short testing duration to maintain tissue hydration Shahmirzadi et al. (2013).

Force (N) and displacement (mm) data were converted into nominal stress and strain values based on sample geometry.

### Existing hyperelastic models

Since urethra tissues undergo large deformations, exhibit nonlinear elastic responses, and are nearly incompressible, hyperelastic models provide a robust theoretical framework to describe their stress–strain behavior. Hyperelasticity is particularly suitable for modeling soft tissues as it is based on a strain energy function $W$, ensuring thermodynamic consistency while capturing large deformation mechanics Holzapfel (2001). For incompressible materials, the Cauchy stress tensor can be expressed as:

4 $$\begin{aligned} \varvec{\sigma } = -p \varvec{I} + 2 \frac{\partial W}{\partial \varvec{C}} \varvec{F} \varvec{F}^T \quad \end{aligned}$$

where: $p$ is the hydrostatic pressure or Lagrange multiplier enforcing the incompressibility constraint, $\varvec{I}$ is the identity tensor, $\varvec{C}$ is the right Cauchy-Green deformation tensor. The right Cauchy-Green deformation tensor $\varvec{C}$ is defined as:

5 $$\begin{aligned} \varvec{C} = \varvec{F}^T \varvec{F} \quad \end{aligned}$$

with $\varvec{F}$ the deformation gradient tensor, which defines the relationship between the reference configuration and the deformed configuration:

6 $$\begin{aligned} \varvec{F} = \frac{\partial \varvec{x}}{\partial \varvec{X}} \end{aligned}$$

$\varvec{X}$ being the position vector in the reference configuration, and $\varvec{x}$ the position vector in the deformed configuration.

From equations 4, 5, and 6, the principal Cauchy stresses $\sigma _i$ can be defined from the strain energy function $W$ as:

7 $$\begin{aligned} \sigma _i = \lambda _i \frac{\partial W}{\partial \lambda _i}, \quad i = 1, 2, 3 \end{aligned}$$

where $\lambda _i$ are the principal stretches. The nominal stress $\sigma n$ also called engineering stress can be obtained from the Cauchy stress and expressed in terms of principal stretches as:

8 $$\begin{aligned} \sigma n_i = \frac{\sigma _i}{ \lambda _i} = \frac{\partial W}{\partial \lambda _i}, \quad i = 1, 2, 3 \end{aligned}$$

Several widely used hyperelastic models in the literature can be applied to biological tissues such as the urethra. Neo-Hookean, Yeoh, Ogden and GOH models were selected in this study because of their ability to progressively capture the increasing complexity of biological tissue behavior, with an emphasis on nonlinear elasticity and fiber reinforcement Parshin et al. (2019); Pawlikowski (2014), making them suitable for representing the mechanical characteristics of the urethra. The following describes the strain energy density function of each model.

The Neo-Hookean model Rivlin (1948) is one of the simplest hyperelastic models. The strain energy density function is given by:

9 $$\begin{aligned} W_{\text {neo}} = C_1 (I_1 - 3) \end{aligned}$$

where $C_1$ is a material constant, and $I_1$ is the first invariant of the right Cauchy-Green tensor, defined as $I_1 = \text {tr}(\textbf{B}) = (\lambda _1^2 + \lambda _2^2 + \lambda _3^2)$. This model is suitable for small to moderate strains but may not accurately capture the behavior of tissues under large deformations. The Yeoh model Yeoh (1993) generalizes the Neo-Hookean model by incorporating higher-order terms in $I_1$:

10 $$\begin{aligned} W = \sum _{n=1}^{3} C_{n} (I_1 - 3)^n \end{aligned}$$

11 $$\begin{aligned} W_{\text {yeoh}} = C_1 (I_1 - 3) + C_2 (I_1 - 3)^2 + C_3 (I_1 - 3)^3 \end{aligned}$$

where $C_1$, $C_2$, and $C_3$ are material constants. This model can capture more complex, nonlinear behaviors observed in biological tissues under large deformations. The Ogden model Ogden (1972) expresses the strain energy density function in terms of the principal stretches $\lambda _i$ and material constants $\mu _n$ and $\alpha _n$:

12 $$\begin{aligned} W_{\text {ogden}} = \sum _{n=1}^{N} \frac{\mu _n}{\alpha _n} \left( \lambda _1^{\alpha _n} + \lambda _2^{\alpha _n} + \lambda _3^{\alpha _n} - 3 \right) \end{aligned}$$

This model is highly flexible and can describe a wide range of nonlinear mechanical behaviors, making it suitable for tissues that undergo significant transverse isotropic deformations. The GOH model Gasser et al. (2006) is designed to account for the anisotropy introduced by fiber orientations within the tissue. The strain energy function in this model is composed of isotropic and transverse isotropic components:

13 $$\begin{aligned} W_{\text {GOH}} = W_{\text {iso}} + W_{\text {aniso}} \end{aligned}$$

where the isotropic part $W_{\text {iso}}$ models the matrix and can be represented by a Neo-Hookean term (equation 9), while the transverse isotropic part $W_{\text {aniso}}$ captures the contribution of fibers:

14 $$\begin{aligned} W_{\text {aniso}}= \frac{k_1}{2 k_2} \sum _{\alpha =1}^{N} (e^{k_2 \left[ \kappa (I_1 - 3) + (1 - 3 \kappa )(I_4^\alpha - 1) - 1 \right] ^2} - 1) \end{aligned}$$

with $k_1 > 0$ a stress-like parameter, $k_2 > 0$ a dimensionless parameter. Those parameters are empirical and not physic-based parameters. $\kappa$ takes into account the dispersion of fiber orientation. N typically represents the number of fiber families or orientations. In this application N=1. The strain invariants $I_1$ and $I_4$ can be written as following wih $A(\alpha )$ is the initial direction of each set of fibers in a fiber-composed material.

15 $$\begin{aligned} I_4(\alpha ) = A(\alpha ) \textbf{F}^T \textbf{F} A(\alpha ) \end{aligned}$$

### Fiber model

The fiber model presented in this paper is inspired by the GOH and Yeoh models, with modifications to the anisotropic component to incorporate physical parameters such as the fiber modulus $E_f$, the fiber volume fraction $\phi$, and an orientation-dependent parameter $\theta$. The model captures the combined effects of the isotropic matrix and the transversely isotropic fibers. The total strain energy density $W_{FM}$ is thus expressed as the sum of the contributions from the matrix and the fibers:

16 $$\begin{aligned} W_{\text {FM}} = (1-\phi )W_{\text {matrix}} + \phi W_{\text {fibers}} \end{aligned}$$

The matrix is described using a Yeoh-like term, which contributes to the strain energy:

17 $$\begin{aligned} W_{\text {matrix}} = C_1 (I_1 - 3) + C_2 (I_1 - 3)^2 + C_3 (I_1 - 3)^3 \end{aligned}$$

The contribution of the fibers was inspired from the anisotropic part of Gasser et al. (2006) represented by an exponential term, which models the nonlinear stiffening response of the fibers as they stretch. To be solved analytically as a 1D problem, the orientation of the fibers have been implemented with a cosinus function such as:

18 $$\begin{aligned} W_{\text {fibers}} = \cos (\theta ) \frac{E_f}{2}\left( \exp \left( k (I_4 - 1)\right) - 1 \right) \end{aligned}$$

where:

- $E_f$ is the fiber modulus, representing the stiffness of the fibers.
- $\phi$ is the volume fraction of fibers in the tissue compared to the matrix.
- $k$ is a parameter that controls the exponential stiffening of the fibers as they are stretched.
- $\theta$ is a parameter representing the orientation of the fibers within the range $[0,\frac{\pi }{2}]$ in our case fibers are supposed aligned following the vector "z" aside the longitudinal direction meaning $\theta =0$ for longitudinal orientation and $\theta =\frac{\pi }{2}$ following circumferential orientation.

**Fig. 4:**
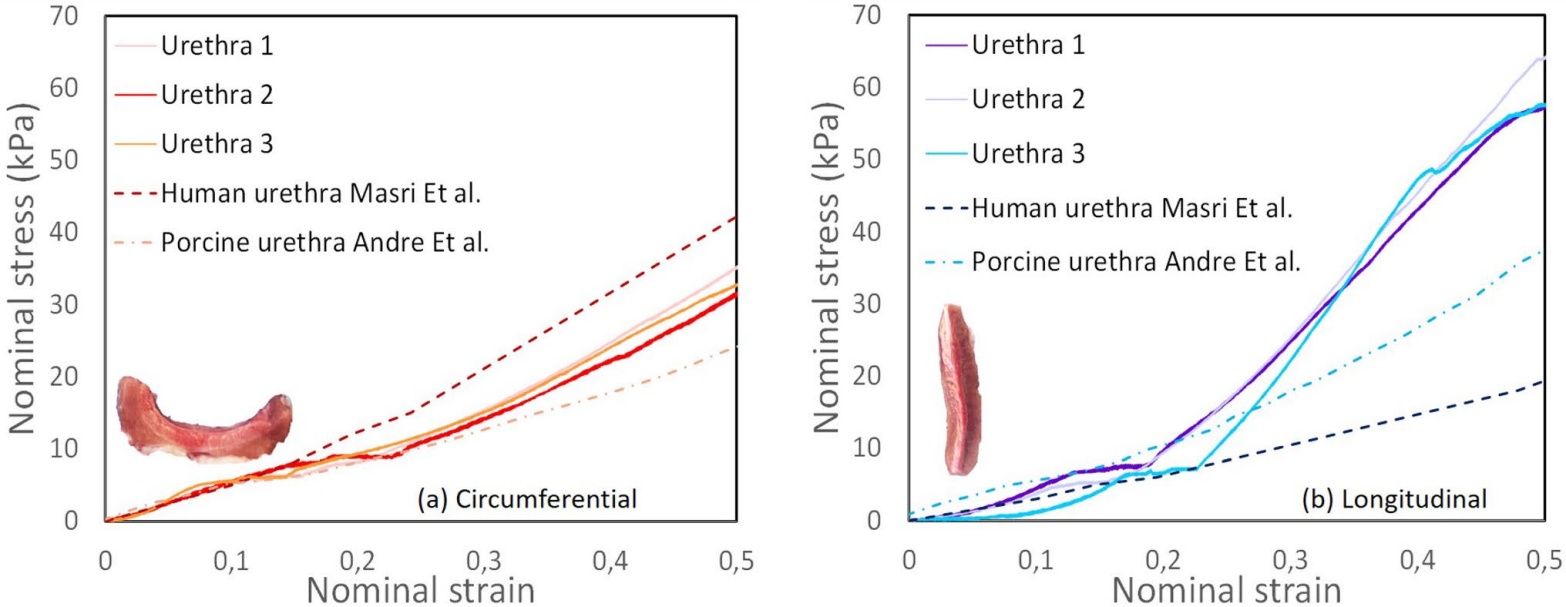
Mean curve of planar tensile tests in uniaxial direction corresponding to the circumferential and longitudinal samples from porcine urethra alongside with porcine urethra planar tensile tests results from André et al. (2022) and human urethra planar tensile tests results from Masri et al. (2018)

Hyperelastic models such as Neo-Hookean, Yeoh, and Ogden are inherently isotropic and do not account for directional anisotropy. Typically, when modeling anisotropic materials, separate parameter sets must be defined for each principal orientation to capture the directional dependence of the mechanical response. In contrast, the GOH model and the fiber-based model proposed in this study inherently incorporate fiber orientation and utilize a single set of parameters to predict the stress–strain relationship for both circumferential and longitudinal directions. To ensure a consistent comparison between models, we defined a single set of parameters for all hyperelastic models presented in Sect. 3.1. This approach allows us to highlight the limitations of isotropic models in capturing the anisotropic behavior of urethral tissue while comparing performances of our fiber-based model with the GOH model.

## Results

This section presents raw data results from aforementioned experiments and will be compared with other porcine urethra results from literature as well as human urethra. Finally, mean values of experimental tests are presented with existing hyperelastic models as well as our own fiber-based model.

### Experimental raw results of porcine urethra

Out of the 18 samples extracted, as described in section 2.4, 12 valid measurements were obtained. For each urethra, two circumferential and two longitudinal samples were analyzed, and the mean stress–strain curve was calculated for each orientation and for each urethra. The nominal stress–strain curves for porcine urethral samples, oriented circumferentially and longitudinally, are shown respectively in Fig. 4(a) and Fig. 4(b). In both cases we plotted alongside our results, experimental data on human urethra from Masri et al. (2018) as well as experimental data on porcine urethra from André et al. (2022). For the circumferentially oriented samples, the individual stress–strain curves for Urethra 1, Urethra 2, and Urethra 3 exhibit consistent mechanical responses, with a gradual increase in nominal stress as strain increases. A nonlinear rise is observed beyond a strain of 0.2, and the mean stress reaches 33.01 kPa at a strain of 0.5. For the longitudinally oriented samples, shown in Fig. 4(b), the initial stress–strain curve exhibits a similar slope compared to circumferentially oriented samples up to 0.25 then a steeper slope appears reaching a stress of 59.4 kPa at a strain of 0.5. The standard deviation for each recorded measurement was computed across the two samples obtained from each urethra in both circumferential and longitudinal orientations. The maximum observed standard deviation was 6.9% for circumferential samples and 8.7% for longitudinal samples. In comparison with the porcine urethral results reported by André et al. (2022), our tested samples generally exhibit higher stress values. The porcine urethras tested by André et al. (2022) reached a stress of 23 kPa at a strain of 0.5 in the circumferential orientation, whereas in the longitudinal orientation, they reached 35 kPa at the same strain level. When comparing with the human urethral samples from Masri et al. (2018), their samples exhibited higher stress in the circumferential orientation, reaching a maximum value of 42 kPa at a strain of 0.5. However, in the longitudinal direction, Masri et al. (2018) reported a lower stress value of 18 kPa at a strain of 0.5, which is significantly lower than the stress observed in our porcine urethral samples. Interestingly, the results from Masri et al. (2018) suggest that the circumferential direction exhibits lower stress than the longitudinal direction, which contrasts with our findings for porcine urethral tissue. Potential explanations for these discrepancies are discussed in Sect. 4. The subsequent section will focus on fitting standard hyperelastic models to the experimental data, with a particular emphasis on incorporating the influence of fibers using our fiber-based model.

### Hyperelastic model comparison

**Fig. 5:**
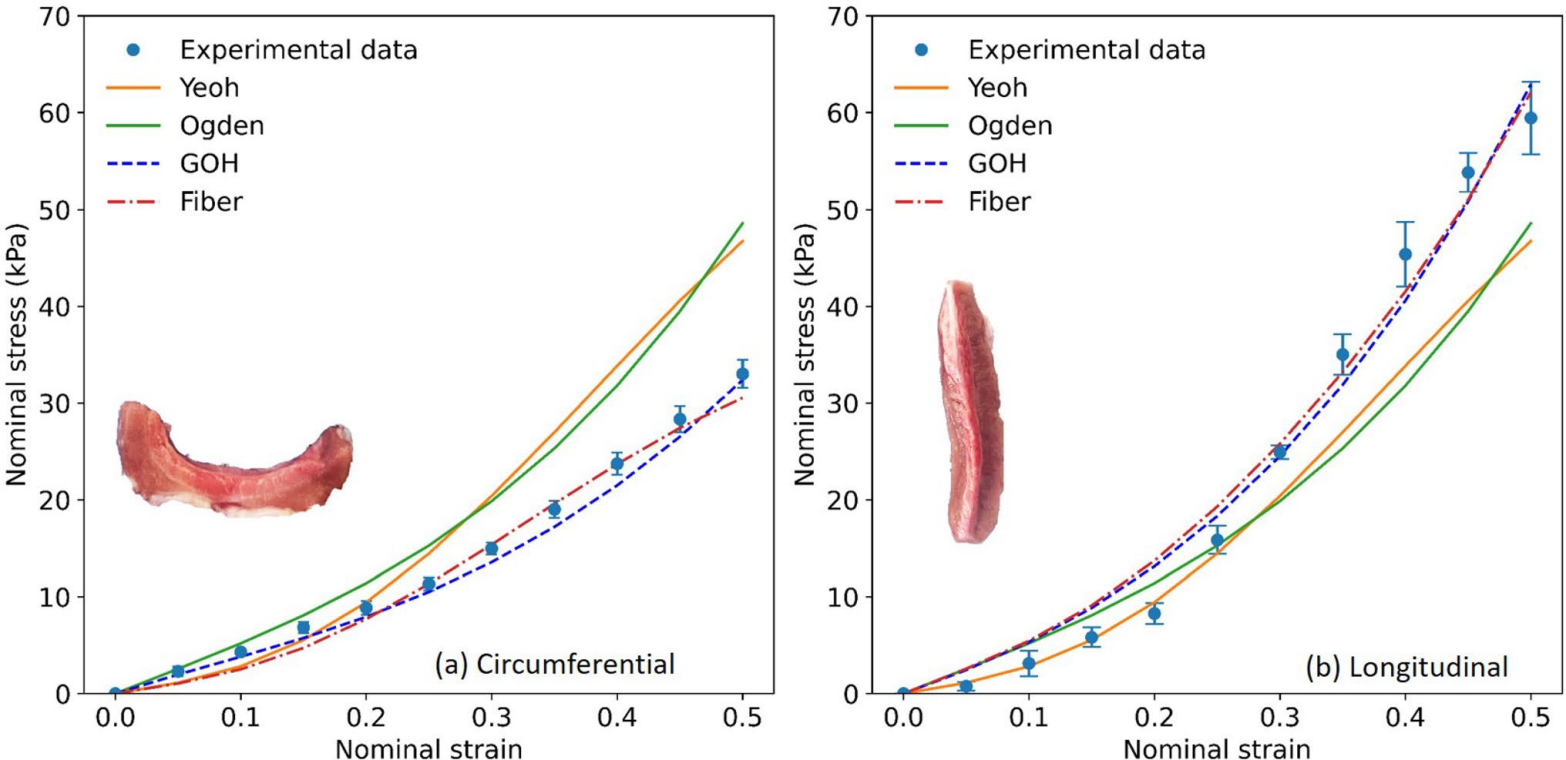
Comparison of hyperelastic models (Yeoh, Ogden, GOH, fiber) alongside with the experimental data mean curve from urethras 1,2,3

To determine the values of the parameters $C_1$, $C_2$, and $C_3$ for the isotropic component ($W_{matrix}$) of our fiber-based model, we adopted an approach similar to the one used by Anssari-Benam et al. (2017). The values of $C_1$, $C_2$, and $C_3$ were initially obtained by fitting the model to deformation data in the circumferential direction, where the fiber contribution is negligible. Subsequently, these parameters were kept fixed while fitting the full model, incorporating the fiber contribution ($W_{fibers}$), to the deformation data obtained from the longitudinal direction.

The performance of the hyperelastic models was evaluated by comparing their fits to the mean experimental curve derived from previously reported data (Fig. 4). The fits for the Yeoh, Ogden, GOH, and fiber models are presented in Fig. 5, with the parameter values summarized in Table 1. Model parameter optimization was carried out using Python 3.12’s minimize function, which employs the L-BFGS-B quasi-Newton algorithm Zhu et al. (1997); Chen et al. (2019).

**Fig. 6:**
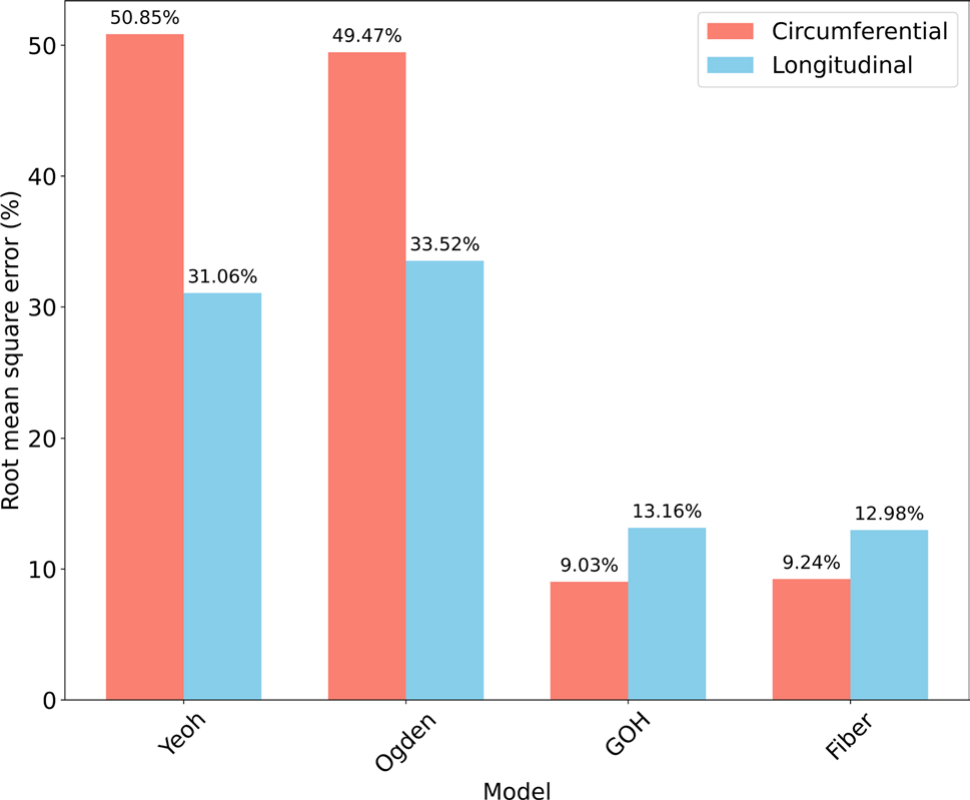
The average root mean square error (RMSE) in percentage for Yeoh, Ogden, GOH and Fiber models compared to experimental data

Both the fiber and GOH models provided accurate fits for the circumferential and longitudinal orientations. In the circumferential direction (Fig. 5(a)), these models effectively captured the sharp increase in stress at high strains, while the Yeoh and Ogden models displayed reasonable fits at small and moderate strains but failed to replicate the tissue stiffening observed at higher deformations. In the longitudinal direction, the fiber and GOH models closely matched the experimental data across the strain range, particularly at large strains. In contrast, the Yeoh and Ogden models performed better at lower strains but diverged significantly at higher strains. Quantitative comparisons were conducted using the root mean square error relative to experimental data for both orientations, as shown in Fig. 6. The fiber model exhibited low RMSE in the circumferential direction (9.24%) and the lowest deviation in the longitudinal direction (12.98%), indicating superior accuracy and consistency. The GOH model also performed well, with a RMSE of 9.01% (circumferential) and 13.16% (longitudinal). In contrast, the Yeoh and Ogden models demonstrated significantly higher values, exceeding 49% circumferentially and 30% longitudinally.

**Table 1:** Hyperelastic material parameters for the different models

Yeoh model		Fiber model	
$C_1$	$3.54$ kPa	$C_1$	$3.54$ kPa
$C_2$	$3.821 \times 10^{1}$ kPa	$C_2$	$3.821 \times 10^{1}$ kPa
$C_3$	$-1.46 \times 10^{1}$ kPa	$C_3$	$-1.46 \times 10^{1}$ kPa
Ogden model		$E_f$	230 MPa
$\mu$	$4.3$ kPa	$\phi$	0.20
$\alpha$	$8$	$\theta$	[0;$\frac{\pi }{2}$] rad
GOH model		*k*	2.02
*C*	$6.91$ kPa		
$k_1$	$1.84 \times 10^{2}$ kPa		
$k_2$	0.9		
$\kappa$	0.3		
$\alpha$	[0;$\frac{\pi }{2}$] rad		

## Discussion

The results presented in Sect. 3 provides valuable insights into the mechanical behavior of porcine urethral tissue and its relevance to understanding human urethral mechanics. Uniaxial tensile testing in both circumferential and longitudinal orientations confirms the anisotropic nature of the porcine urethra, with distinct mechanical responses depending on the loading direction. This anisotropy, which is assumed to be transversely isotropic, aligns with histological findings (Sect. 2.3), where type I collagen fibers are predominantly oriented along the longitudinal axis. This structural organization results in greater stiffness in the longitudinal direction, where fibers bear a higher load, while the circumferential direction exhibits lower stiffness due to reduced fiber contributions. When comparing our findings with previous studies on porcine urethras by André et al. (2022), we observe that in the circumferential orientation, our results are close, showing a slightly higher stress–strain response. However, in the longitudinal orientation, notable differences arise, with our data indicating higher stress values. These discrepancies may be attributed to variations in sample preparation, differences in testing protocols, or interspecies variability Cunnane et al. (2021a). Nonetheless, our observation of higher stiffness in the longitudinal direction compared to the circumferential direction is consistent with the findings reported by André et al. (2022). When comparing our results with human urethral tissue data from Masri et al. (2018), we find that porcine urethral tissue exhibits higher stress values at a given strain level, particularly in the longitudinal direction, suggesting increased stiffness at higher strains. This difference may be due to species-specific variations in fiber geometry, collagen content, and microstructural organization. Despite these variations, the low standard deviation observed across our porcine samples indicates that the mechanical behavior is reproducible. However, due to the limited number of urethral samples tested, a larger study would be necessary to improve the statistical reliability and generalizability of these findings.

Interestingly, the results from Masri et al. suggest that the circumferential direction exhibits lower stress than the longitudinal direction, which contrasts with typical expectations based on the histological organization of urethral tissue Natali et al. (2016). This discrepancy may stem from differences in testing conditions, sample preservation techniques, or variations in collagen fiber orientation and elastin content Cunnane et al. (2021b).

The fiber-based hyperelastic model developed in this study provides improved accuracy in predicting urethral tissue mechanics, particularly when compared to traditional hyperelastic models such as Yeoh and Ogden. These conventional models, which lack inherent anisotropic considerations, require separate parameter adjustments for each loading direction to achieve reasonable fits. By incorporating fiber contribution, our fiber-based model captures the anisotropic response more effectively, particularly at higher strain levels. The Gasser-Ogden-Holzapfel (GOH) model also performs well in characterizing urethral mechanics; however such a complex definition have been simplified to get only meaningful measurable parameter such as fiber volume fraction, elastic modulus and orientation. Despite the accuracy and relevance of the proposed fiber model, several limitations should be acknowledged. First, this approach, giving fast efficient results, is suitable only for analytical solving problems. Second, the study was limited to three porcine urethras and a total of 12 samples tested, as acquiring fresh animal tissue remains a significant challenge. While our results are consistent with previous mechanical characterizations of urethral tissue, further analyses involving larger sample sizes would enhance the robustness of our findings. Moreover, while this research contributes to the design and optimization of artificial urinary sphincters (AUSs), the strain levels tested (up to 0.5) may not fully capture the complete mechanical response of the porcine urethra under physiological conditions. Future investigations should aim to extend the strain range, particularly under higher deformation scenarios.

## Conclusion

This study provides a comprehensive mechanical characterisation of porcine urethral tissues, highlighting their anisotropic behavior through uniaxial tensile tests. The findings confirm the relevance of porcine urethra as a replacement for human urethral mechanics, although some differences in mechanical properties appear. By introducing a fiber-based model that integrates physical parameters such as fiber modulus, volume fraction, and fiber orientation, we fit the mechanical response of the tissue under physiologically relevant conditions. This model requires only one set of parameters to predict both circumferential and longitudinal orientation, demonstrating good accuracy. These insights contribute to the refinement of constitutive models for fiber-composed soft tissues, bringing the physical parameters consideration not always present in existing models. The accuracy of our fiber model underscores the necessity of considering physically meaningful parameters and understanding the microstructural complexity of biological tissues when designing artificial urinary sphincters (AUS) and other medical devices general. Future developments should incorporate fiber geometry and layer-specific orientation, advancing toward a multi-scale modeling approach. Such approach would enable the prediction of mechanical properties that are difficult to measure experimentally. Moreover, the fiber model developed in this study could be extended to other fiber-reinforced soft tissues, enhancing its applicability to three-dimensional modeling and applications beyond urethral mechanics.
